# Regulatory role of Chitinase 3-like 1 gene in papillary thyroid carcinoma proved by integration analyses of single-cell sequencing with cohort and experimental validations

**DOI:** 10.1186/s12935-023-02987-7

**Published:** 2023-07-21

**Authors:** Xiaojun Zhang, Wanwan Peng, Jie Fan, Ruihua Luo, Shanting Liu, Wei Du, Chaochao Luo, Jiawen Zheng, Xinghua Pan, Hong Ge

**Affiliations:** 1 Department of Head Neck and Thyroid Surgery, The Affiliated Cancer Hospital of Zhengzhou University & Henan Cancer Hospital, 450008 Zhengzhou, China; 2 Department of Biochemistry and Molecular Biology, School of Basic Medical Sciences, Guangdong Provincial Key Laboratory of Single Cell Technology and Application, Southern Medical University, 510515 Guangzhou, China; 3 Department of Molecular Pathology, The Affiliated Cancer Hospital of Zhengzhou University & Henan Cancer Hospital, 450008 Zhengzhou, China; 4 Department of Pediatrics, Nanfang Hospital, Southern Medical University, 510515 Guangzhou, China; 5 Department of Hepatobiliary Surgery II, Zhujiang Hospital, Southern Medical University, 510515 Guangzhou, China; 6 Department of Radiation Oncology, The Affiliated Cancer Hospital of Zhengzhou University & Henan Cancer Hospital, 450008 Zhengzhou, China

**Keywords:** Single-cell RNA sequencing, Papillary thyroid carcinoma, CHI3L1, Invasion and metastasis, Subpopulation

## Abstract

Papillary thyroid carcinoma (PTC) is one of the most common thyroid carcinomas. The gross extrathyroidal extension and extensive metastases of PTC lead to high rates of recurrence and poor clinical outcomes. However, the mechanisms underlying PTC development are poorly understood. In this study, using single-cell RNA sequencing, the transcriptome profiles of two PTC patients were addressed, including PTC1 with low malignancy and good prognosis and PTC2 with high malignancy and poor prognosis. We found that epithelial subcluster Epi02 was the most associated with the malignant development of PTC cells, with which the fold change of Chitinase 3-like 1 (CHI3L1) is on the top of the differentially expressed genes between PTC1 and PTC2 (*P* < 0.001). However CHI3L1 is rarely investigated in PTC as far. We then studied its role in PTC with a series of experiments. Firstly, qRT-PCR analysis of 14 PTC patients showed that the expression of CHI3L1 was positively correlated with malignancy. In addition, overexpression or silencing of CHI3L1 in TPC-1 cells, a PTC cell line, cultured in vitro showed that the proliferation, invasion, and metastasis of the cells were promoted or alleviated by CHI3L1. Further, immunohistochemistry analysis of 110 PTC cases revealed a significant relationship between CHI3L1 protein expression and PTC progression, especially the T (*P* < 0.001), N (*P* < 0.001), M stages (*P* = 0.007) and gross ETE (*P* < 0.001). Together, our results prove that CHI3L1 is a positive regulator of malignant development of PTC, and it promotes proliferation, invasion, and metastasis of PTC cells. Our study improves understanding of the molecular mechanisms underlying the progression of PTC and provides new insights for the clinical diagnosis and treatment of PTC.

**Supplementary Information:**

The online version contains supplementary material available at 10.1186/s12935-023-02987-7.

## Introduction

Thyroid cancer is the most common endocrine malignancy. Papillary thyroid cancer (PTC) accounts for about 85% of all thyroid cancers [1, 2]. Although over 95% of PTC patients are curable and achieve 10-year survival [3], some of them have an aggressive phenotype associated with malignant clinical outcomes because of comprehensive lymph node metastasis, invasion of extranodal tissues, or even distant metastasis [4–6]. Studies have shown that lymph node metastasis in elderly thyroid cancer patients can lead to an even worse prognosis [7–9], but the specific underlying mechanism is unclear. Single-cell RNA sequencing (scRNA-seq) analysis of PTC tissue revealed differences between genders [10], the evolutionary relationship between PTC and ATC [11], the tumor microenvironment associated with PTC [12], and differences in the nature of metastatic and non-metastatic PTC cells, but the molecular regulatory mechanism remains to be fully elucidated.

Transcriptome changes of the disease can be captured at single-cell resolution [13, 14]. scRNA-seq technology has developed rapidly and has been applied in various fields [15–17], especially in the study of tumor heterogeneity, differentiation, metastasis, and microenvironment [18–21]. In this study, we performed scRNA-seq to analyze 3,497 cells from tumor tissues derived from two PTC patients with different malignancies. We analyzed the transcriptional features, cell trajectories, and cell–cell crosstalk in PTCs, and found that chitinase 3-like 1 (CHI3L1) is an important gene regulating the malignancy of PTC. CHI3L1 is found to be involved in the malignancy of breast cancer, melanoma and lung cancer, etc. [22–24]. Two recent studies find that overexpression of CHI3L1 protein is related to PTC with lymph node metastases [25, 26]. However, the role and the mechanism of CHI3L1 on PTC remains to be validated. We used TPC-1 cells to verify the function of CHI3L1 in regulating cell proliferation, invasion, and metastasis in vitro. We also validated the findings in a clinical cohort of 110 PTC patients as well as a set of gene-overexpressing experiments in a cell line model for the functional significance of CHI3L1 in PTC. Our results not only deepen our understanding of the molecular regulatory mechanism of metastasis of PTC, but also provide new evidence for the clinical diagnosis and selection of therapeutic targets for PTC treatment, potentially promoting precision medicine for PTC.

## Materials and methods

### Sample collection and clinical information

Two representative PTC patient biopsies (PTC1 and PTC2) were obtained from the department of Head Neck and Thyroid Surgery, The Affiliated Cancer Hospital of Zhengzhou University. Both patients were independently diagnosed by two experienced pathologists. Biopsy histology analysis was conducted in a blinded manner; the diagnosis was confirmed and the tissues were classified according to the eighth edition of the American Joint Committee on Cancer Tumor Node Metastasis classification (AJCC TNM) system. Informed consent was obtained before biopsy collection. The experiments were approved by the Ethics Committee (JS-1491). For PTC1, cervical lymph node metastases were detected, but the patient recovered after surgical resection. For PTC2, the primary lesions invaded the trachea and larynx, and cervical and mediastinal lymph node metastases, lung metastases, and bone metastases were detected, none of which were cured (Table S1).

### Preparation of single-cell suspensions, single-cell RNA seq and data procession

Fresh tumor tissues were cut into fragments which were followed by a series of processes to form the single-cell suspensions (Details seen in Supplementary 1.1).

Single cells were captured and cDNA libraries were generated using the V3 Gel Bead Kit (10x Genomics, Pleasanton, CA) according to standard protocols. The cDNA libraries were sequenced on an Illumina HiSeq 4000 with paired-end 150-bp reads. The genetic barcode matrix was generated by the Cell Ranger Toolkit (v3.1), where the droplet-based sequencing data were aligned with the GRCh38 human reference genome, and the number of unique molecular identifiers (UMIs) was abstracted for each transcript in each cell. Quality control and batch effect correction of scRNA-seq data were detailed in Supplementary 1.2. Dimensional reduction and cellular annotation were detailed in Supplementary 1.3.

### Analysis of cell–cell communication analysis and copy number variation evaluation

CellPhoneDB (https://github.com/Teichlab/cellphonedb) was used to perform the cell–cell communication analysis. Ligands that belong to the cheetah and growth families with *P* < 0.05 were used to evaluate the relationships between cell clusters.

The Copycat R package was used to detect aneuploid cells in PTC cells based on single-cell RNA-seq raw counts and default parameters.

### Analysis of cell trajectories and SCENIC analysis

Trajectory analysis was performed using the Monocle 3 package to reveal all epithelial cell state transitions from PTC. The integrated expression matrix with batch effect removed was used as the input data, and the cell trajectory and order were inferred with default settings.

SCENIC analysis was conducted as described previously. We used the pySCENIC package (version 0.11.2), a lightning-fast python implementation of the SCENIC pipeline. Two gene motif rankings (10 kb around the transcription start site [TSS] or 500 bp upstream of the TSS) were used to determine the search space around the TSS, and the 20-thousand motif database was used for RcisTarget and GENIE3.

### Gene set enrichment analysis (GSEA)

To investigate the functional network of genes that are differentially expressed in epithelial subclusters, gene set enrichment analysis (GSEA) was used to identify the gene sets that are enriched in either cluster of epithelial cells, with the following criteria: *P* < 0.05 and false discovery rate q < 0.25.

( http://bioinfo.life.hust.edu.cn/GSCA/#/; https://cistrome.shinyapps.io/timer/)

### Construction of overexpression vector and short hairpin RNA vector

The human PTC cell line TPC-1 was gifted from the Center for Reproductive Medicine, First Affiliated Hospital, Sun Yat-sen University. The CHI3L1 overexpression plasmid was constructed as previously reported [27]. Briefly, total RNA was extracted from TPC-1 cells using the RNAeasy kit (Qiagen) and cDNAs were synthesized using PrimeSTAR Max Premix (2×) (Takara R045A). The coding sequence of CHI3L1 was amplified by PCR using CHI3L1-specific primers. The primer sequences are shown in Table S2. The product was subcloned into the eukaryotic expression vector pCDNA3.1-3xflag-C (FITGENE, China). The pCDNA3.1-3xflag vector was used as an empty vector (pCDNAflag) control.

The CHI3L1 short hairpin RNA (shRNA) plasmid was constructed as previously reported [28]. The primer sequences to generate the CHI3L1 shRNA vector are shown in Table S3. The product was subcloned into the eukaryotic expression vector pmRZip (FITGENE, China). The silencing efficiency of CHI3L1 shRNA was determined by RT-PCR.

### RNA extraction and qRT-PCR

RNA was extracted from cells and tissues with the RNAeasy kit (Qiagen). cDNA was prepared (miScript II RT kit) and used for qPCR, and the results were normalized to actin levels. The primer sequences are shown in Table S4.

### Western blot, cell proliferation, cell migration and invasion analysis

Western blot was detailed in Supplementary 1.4. The proliferation, migration and invasion of TPC-1 cells were tested by CCK8 assay, Transwell migration assay and Matrigel-coated Transwell chambers, respectively (Details seen in Supplementary 1.5).

### Statistical analysis

Results are reported as mean ± standard deviation (n = 3). Data were analyzed using R 4.0.3 and statistical significance between groups was analyzed with one-way ANOVA. *P* < 0.05 was considered statistically significant. Western blot protein band intensities were evaluated using ImageJ.

## Results

### Subsection

#### Single-cell transcriptomic landscape of PTC cells

**Fig. 1:**
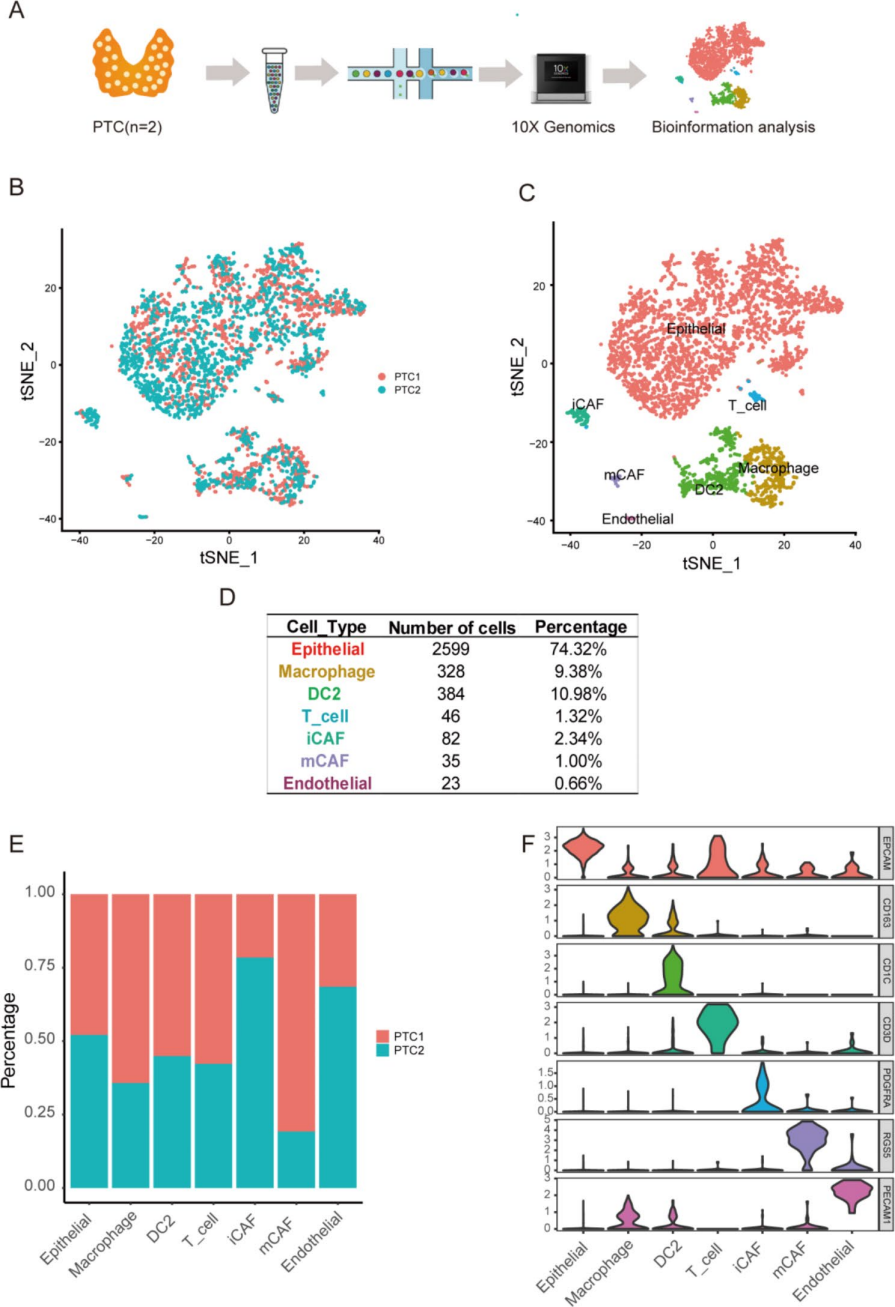
Transcriptome profile of PTC at the single-cell level. (**A**) Process and principle of the experiment. (**B**) t-SNE plot of all high-quality cells colored by major cell lineage. (**C**) t-SNE plot of each subpopulation in PTC1 and PTC2. (**D**) Proportion of the subpopulations in the silico combination of the data from the two PTC patients. (**E**) Proportion of each subpopulation in PTC1 and PTC2. (**F**) Major marker genes for each subpopulation

To explore the cellular transcriptomic atlas of PTC, scRNA-seq analysis of two biopsies from different patients was conducted by 10x Genomics (Fig. 1A). After quality control, we obtained a total of 3,497 single-cell transcriptomes (1,705 for PTC1, 1,792 for PTC2). T-distributed stochastic neighbor embedding (t-SNE) visualization of the cells revealed seven main clusters. Based on the expression of marker genes, the seven clusters were annotated as epithelial cells, macrophages, dendritic cells 2 (DC2s), T cells, inflammatory cancer-associated fibroblasts (iCAFs), myo-cancer-associated fibroblasts (mCAFs), and endothelial cells (Fig. 1B). We observed that each of the seven clusters was present in both samples (Fig. 1C).

Among these clusters, the epithelial cluster was the dominant cluster, covering about 75% of the total number of cells obtained (Fig. 1D). The numbers of iCAFs and endothelial cells were highest in PTC2, and the numbers of macrophages and mCAFs were highest in PTC1 (Fig. 1E). The numbers of epithelial cells, T cells, and DC2s were not different between PTC1 and PTC2 (Fig. 1E). The expression levels of some marker genes, such as epithelial cell adhesion molecule (EPCAM), CD163, CD1C, CD3D, platelet-derived growth factor receptor alpha (PDGFRA), regulator of G protein signaling 5 (RGS5), and platelet endothelial cell adhesion molecule-1 (PECAM1), were carefully checked in these clusters. The results showed that, characteristically, EPCAM was highly expressed in epithelial cells and T cells, CD163 was highly expressed in macrophages, CD1C was highly expressed in DC2s, CD3D was highly expressed in T cells, PDGFRA was highly expressed in iCAFs, RGS5 was highly expressed in mCAFs, and PECAM1 was highly expressed in endothelial cells (Fig. 1F).

### Function and copy number variation (CNV) profile of PTC cells

**Fig. 2:**
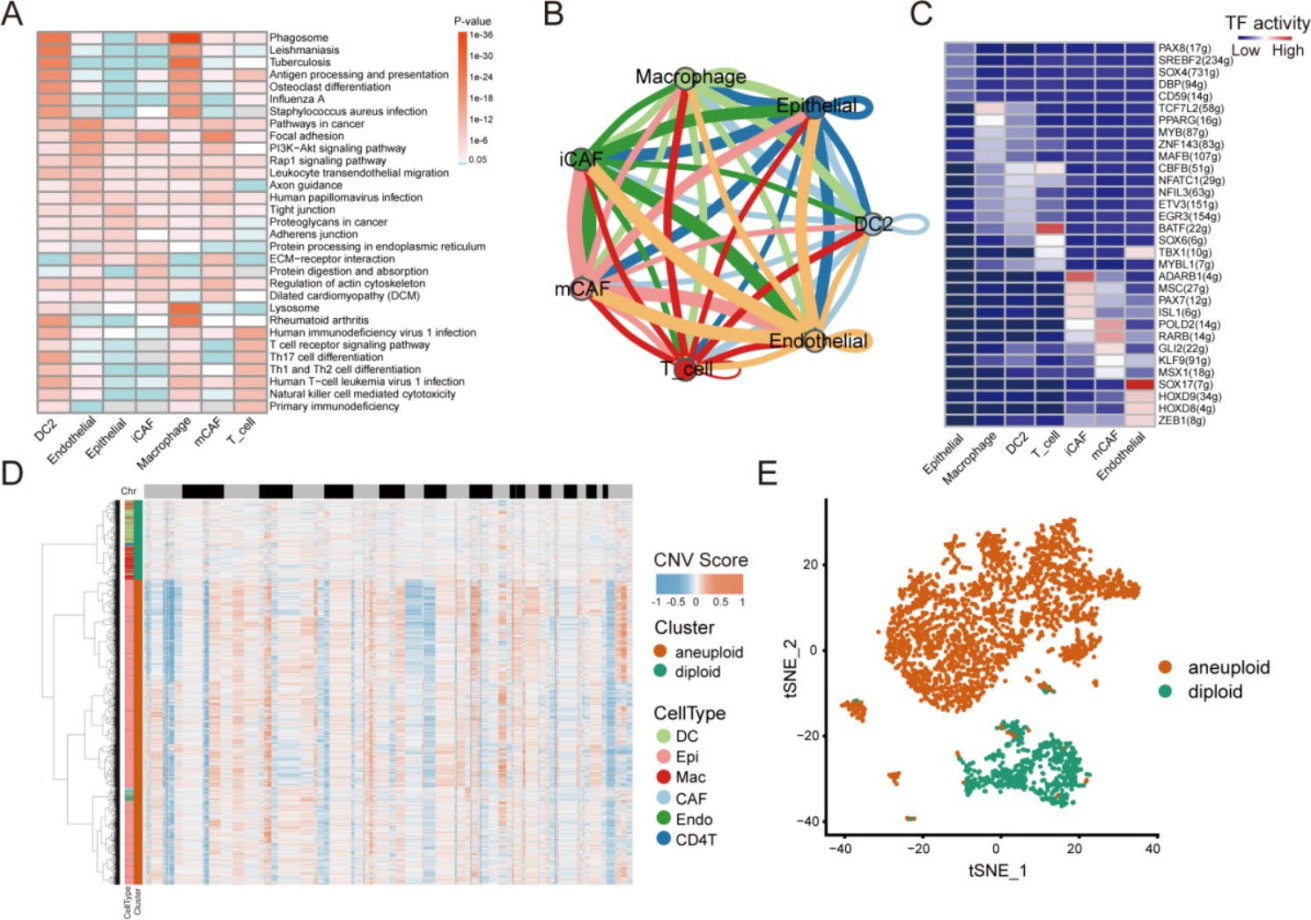
Function and copy number variation (CNV) analysis of PTC. (**A**) Pathway enrichment of marker genes for the major cell clusters. (**B**) Cell communication among the major cell clusters. (**C**) Transcription factor activity based on the top 5 activated transcription factors in each major cluster. (**D**-**E**) The RNA inferred copy number variation (inferCNV) of individual cells

To investigate the functions of these cell clusters in PTC cells, the associated molecular pathways were investigated. The enrichment analysis of marker genes of each cluster showed that the signaling pathways with the most abundant genes in each cluster were different (Fig. 2A). The signaling pathways related to Phagosome, antigen processing and presentation, Th17 cell differentiation, and human T cell leukemia virus 1 infection were the top pathways in the DC2 cluster. The signaling pathways of cancer, focal adhesion, PI3K-Akt, rap1, leukocyte transendothelial migration, tight junction, and proteoglycans were the top pathways in the endothelial cell, epithelial cell, iCAF, and mCAF clusters. The signaling pathway of T cell receptor, Th17 cell differentiation, Th1 and Th2 cell differentiation, human T cell leukemia virus 1 infection, natural killer cell-mediated cytotoxicity, and primary immunodeficiency were the top pathways in the T cell cluster (Fig. 2A).

To explore the interaction network among different clusters in PTC cells, cell communication of these clusters was analyzed based on potential ligand-receptor pairs in cells. The results revealed that there were extensive communications between these clusters (Fig. 2B). The epithelial cell cluster had an apparent interaction with the macrophage, iCAF, mCAF, and endothelial cell clusters, and so did the DC2 cluster with the macrophage cluster. Interactions were also detected between the macrophage and the epithelial cell and DC2 clusters; iCAF and epithelial cell clusters; mCAF and endothelial clusters; mCAF and epithelial cell clusters; iCAF and endothelial cell clusters; endothelial cluster and epithelial cell clusters; and iCAF and mCAF clusters (Fig. 2B).

To explore the regulatory function of each cluster in PTC cells, transcription factor activity was analyzed based on the top 5 activated transcription factors in each cluster. The results showed that the activated transcription factors in each cluster were different (Fig. 2C). TCF7L2 was highly activated in the macrophage cluster; BATF and CBFB were highly activated in the T cell cluster; ADARB1, MSC, PAX7, and ISL1 were highly activated in the iCAF cluster; POLD2, RARB, and GLI2 were highly activated in the mCAF cluster; and SOX17, HOXD9, HOXD8, ZEB1, and TBX1 were highly activated in the endothelial cell cluster. In the epithelial cell and DC2 clusters, no transcription factors were highly activated (Fig. 2C).

To identify the differences in the genomic profile of each cluster in PTC cells, the inferred copy number variation (inferCNV) was analyzed based on the scRNA-seq data without a specifically defined reference. The results showed that a higher CNV burden (aneuploid) was identified in the epithelial cell, iCAF, T cell, and mCAF clusters and a lower CNV burden (diploid) was identified in the DC2 and macrophage clusters (Fig. 2D-E).

### Molecular signature of the epithelial cluster of PTC cells

**Fig. 3:**
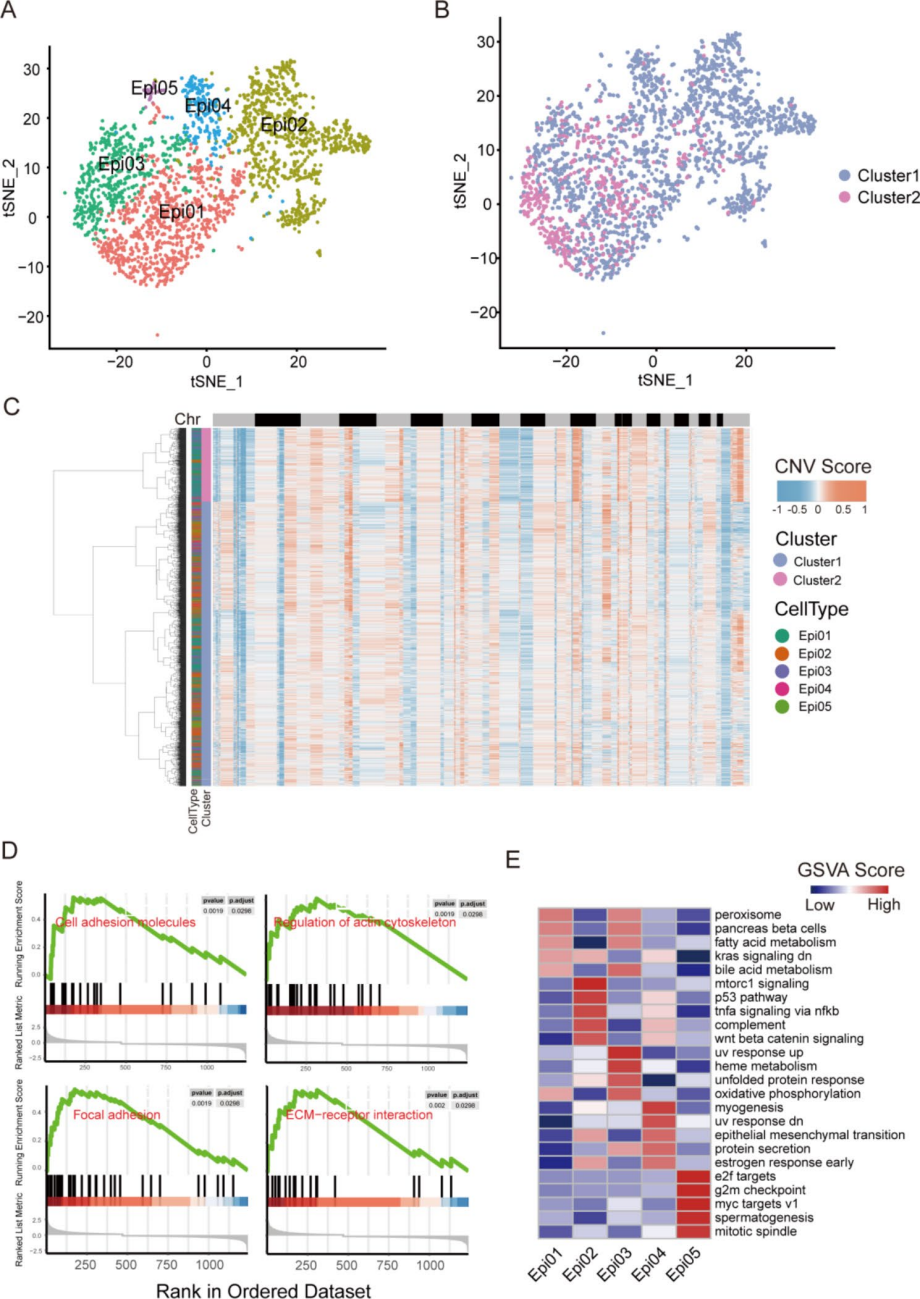
Molecular signature of epithelial clusters of PTC. (**A**) t-SNE plot of all epithelial cells profiled in the present study colored by major cell lineage. (**B**-**C**) RNA inferred copy number variation (inferCNV) of the individual cells; two clusters are demonstrated based on inferCNV pattern. (**D**-**E**) GSEA of marker genes for the major lineages of the epithelial cells

Because the epithelial cluster was the dominant cluster and a higher inferCNV burden was identified in this cluster, we deduced that this cluster was comprised of the PTC cells and analyzed its molecular signature. The t-SNE visualization of these cells revealed five subclusters (Epi01-Epi05), based on the overall profile of gene expression (Fig. 3A). On the other hand, the inferCNV analysis revealed two CNV clusters (CNV clusters 1 and 2), both of which were present in every subcluster of the epithelial cluster (Fig. 3B). In the five subclusters, Epi01 and Epi03 were mainly enriched in the low-CNV cluster (cluster 2) and Epi02, Epi04, and Epi05 were mainly enriched in the high-CNV cluster (cluster 1) (Fig. 3B-C). These results indicate heterogeneity between tumor cells.

To investigate the functions and pathways of each subcluster, GSEA was conducted. The results revealed that these five subclusters were associated with cell adhesion molecules, regulation of the actin cytoskeleton, focal adhesion, and extracellular matrix-receptor interaction (Fig. 3D). However, the enriched pathways were different in different subclusters. Epi01 was enriched in the peroxisome, pancreas beta cells, fatty acid metabolism, kras signaling (dn), bile acid metabolism, and oxidative phosphorylation pathways. Epi02 was enriched in mTORC1 signaling, p53 pathway, TNF-α signaling via NF-κB, complement, Wnt beta catenin signaling, KRAS signaling (dn), epithelial-mesenchymal transition, estrogen response and the unfolded protein response. Epi03 was enriched in the UV response (up), heme metabolism, unfolded protein response, oxidative phosphorylation, protein secretion, bile acid metabolism, peroxisome, pancreas beta cells, and fatty acid metabolism pathways. Epi04 was enriched in the myogenesis, UV response (dn), epithelial–mesenchymal transition, protein secretion, early estrogen response, p53 pathway, TNF-α signaling via NF-κB, complement, Wnt β-catenin signaling, and kras signaling (dn) pathways. Epi05 was enriched in the E2f targets, G2M checkpoint, myc targets v1, spermatogenesis, and mitotic spindle pathways (Fig. 3E). The Epi02 subcluster was most enriched in recognized cancer pathways, such as mTORC1 signaling, the p53 pathway, and kras signaling (dn), which indicates a potential role of Epi02 in PTC metastasis.

### Epi02 epithelial subcluster most associated with the malignant development of PTC cells

**Fig. 4:**
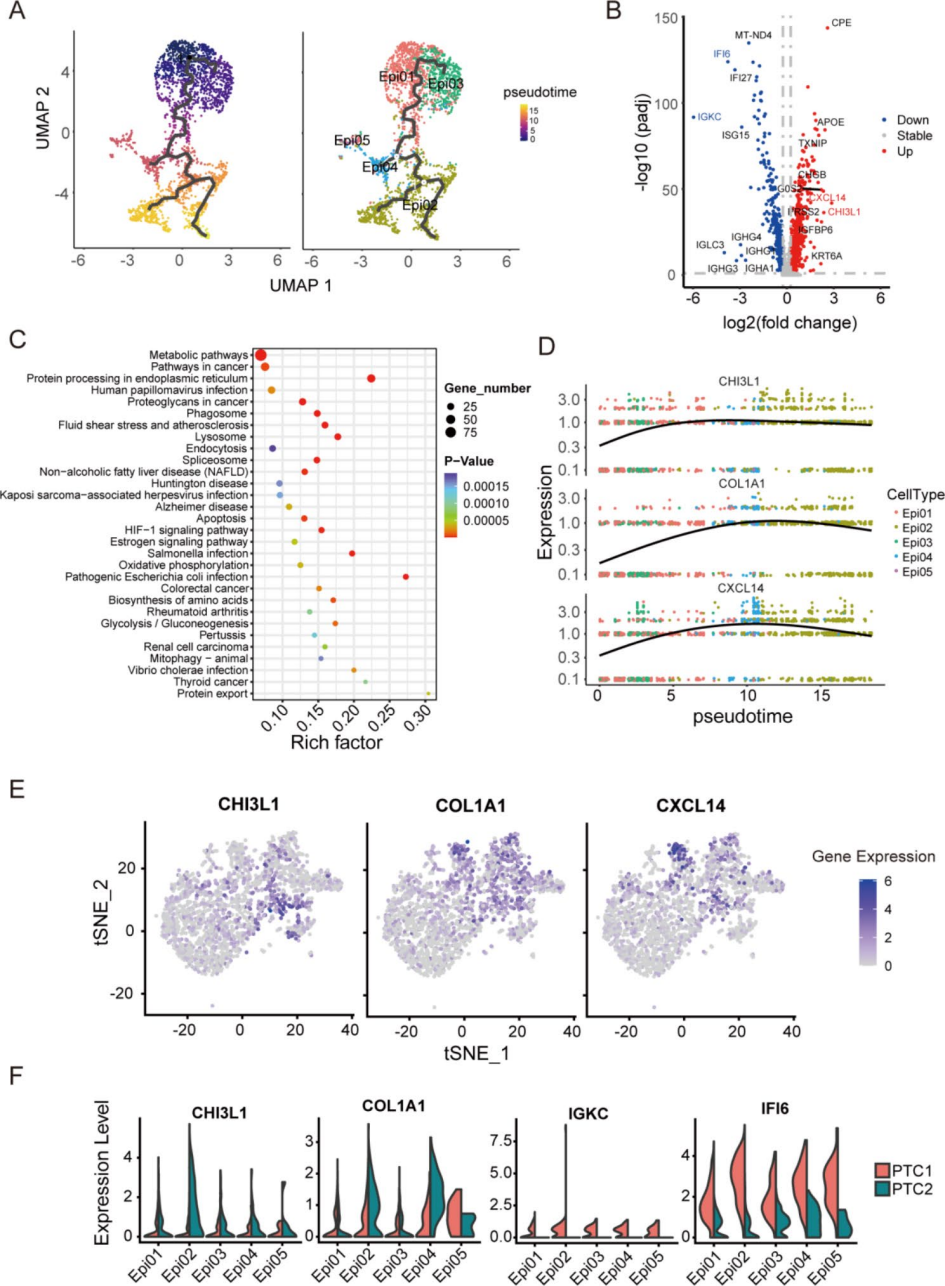
The Epi02 epithelial subcluster stands as one of the important cell clusters for malignant development of PTC. (**B**, **C**) Subcluster Epi02. (**A**, **D**, **E**, **F**) All epithelial lineages covering all five epithelial subclusters. (**A**) Pseudotime trajectory analysis of all five epithelial subclusters. (**B**) The fold change value of CHI3L1 is highest among differentially expressed genes in epithelial subcluster Epi02 between PTC1 and PTC2. (**C**) KEGG pathway analysis of different genes in epithelial subcluster Epi02 between PTC1 and PTC2. (**D**-**F**) The expression of CHI3L1, COL1A1, and CXCL14 in epithelial lineages between PTC1 and PTC2

The differentially expressed genes (DEGs) in epithelial subcluster 2 between PTC1 and PTC2 were obtained. The results showed that the expression of some tumor-related genes, including CHI3L1, collagen 1A1 (COL1A1), and C-X-C motif chemokine ligand 14 (CXCL14), in PTC2 was significantly higher than that in PTC1, but the expression of some immune-related genes, such as IGH, IGKC, IFI6, and IFI27, in PTC2 was significantly lower than that in PTC1 (Fig. 4B). The functions of the DEGs in epithelial subcluster 2 between PTC1 and PTC2 were analyzed, and the top 30 pathways are shown in Fig. 4C. The results show that some pathways associated with carcinogenesis, such as the HIF-1 signaling pathway and the thyroid cancer pathway, were enriched in upregulated genes in PTC2 (Fig. 4C). However, immune evasion may have been present during the process of invasion and metastasis of PTC.

To determine the development of epithelial cells in PTC, we performed a pseudotime trajectory analysis of all five subclusters of epithelial cells (Fig. 4A). The expression of three tumor-related genes among the five subclusters was dissected. The results show that the expression of CHI3L1, COL1A1, and CXCL14 was lower in Epi01 and Epi03 (at the beginning of the pseudotime trajectory) and higher in Epi02 (at the end of the pseudotime trajectory) (Fig. 4D-E). CHI3L1, COL1A1, and CXCL14 have been shown to be related to tumor metastasis and invasion [29–32]. This result suggests that COL1A1 and CXCL14 are potential regulatory factors of metastasis and the malignant development of PTC.

Early studies have shown that CHI3L1 is highly expressed in a variety of tumor tissues, such as gastric cancer, lung cancer, and ovarian cancer [33]. We further analyzed the expression of CHI3L1 in the five subclusters of epithelial cells between PTC1 and PTC2. The results showed that in Epi01 and Epi03, the expression of CHI3L1 was not significantly different between PTC1 and PTC2, but in Epi04, Epi05, and Epi02; the expression of CHI3L1 was significantly higher in PTC2 than in PTC1, and particularly in Epi02, the difference was the greatest (Fig. 4F). This suggests that Epi02 is the subcluster most associated with PTC metastasis.

### Association of CHI3L1 with the invasion and metastasis of a variety of other malignant tumors

**Fig. 5:**
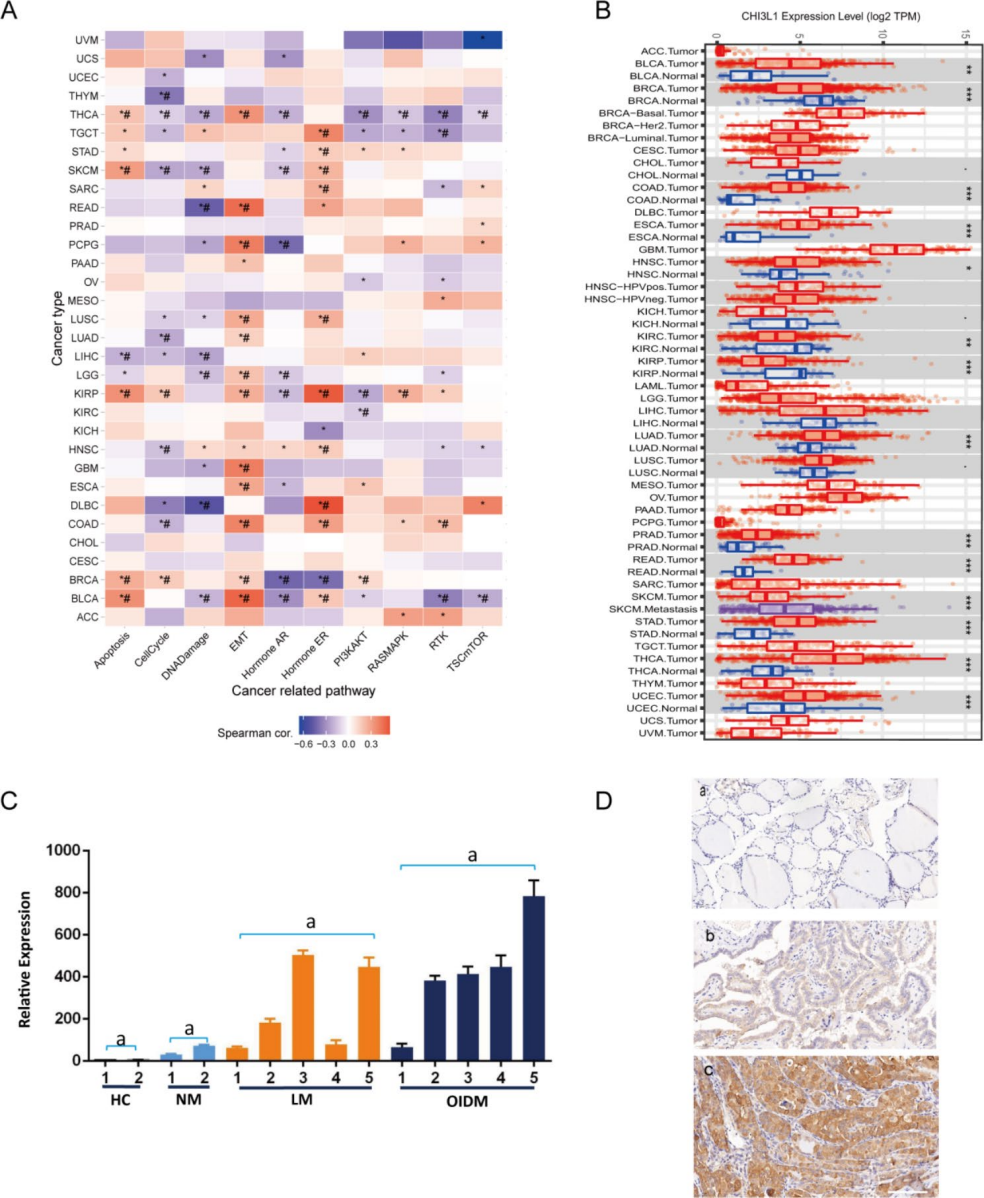
CHI3L1 is associated with tumor invasion and metastasis. (**A**) CHI3L1 mRNA is highly expressed in a variety of malignant tumors. (**B**) Correlation analysis of CHI3L1 and activated tumor-associated signaling pathways in different malignant tumors. (**C**) Expression of CHI3L1 in PTC clinical biopsies with different degrees of malignancy. HC, healthy control; NM, no metastasis (TNM stage T1N0, T2N0); LM, general metastasis (TNM stage T1N1a, T3N1a, T3bN1b, T4aN1b, T4bN1b); OIDM, obvious invasion and distant metastasis (TNM stage T4N1bM1), different lowercase letters indicate significant differences (*P* < 0.05). (**D**) Expression of CHI3L1 in normal thyroid tissue as control (**a**), PTC with general metastasis (**b**) and PTC with obvious invasion and distant metastasis (**c**) as analyzed by immunohistochemistry. Scale bar, 50 μm

Aiming at CHI3L1, the highest fold change value among differentially expressed genes in the highly malignant cell subsets found by scRNA-seq analysis in the clinical PTC tissues, we further performed GSEA based on the TCGA database and found that CHI3L1 mRNA was highly expressed in various malignant tumors such as bladder urothelial carcinoma, colon cancer, esophageal cancer, breast invasive carcinoma, head and neck squamous cell carcinoma, renal clear cell carcinoma, and lung adenocarcinoma (Fig. 5A). We also found that CHI3L1 is involved in various malignant tumor-associated signaling pathways (Fig. 5B), among which epithelial-mesenchymal transition, PI3K/AKT, RAS/MAPK, and TSC-mTOR were associated with tumor migration and invasion. These results above indicate that CHI3L1 is related to the invasion and metastasis of malignant tumors.

### Investigation of CHI3L1 expression pattern in a cohort of PTC patients

CHI3L1 was one of the representative genes expressed in the more malignant cell subsets and the more malignant patient PTC2, as revealed by the analyses above. We obtained a set of biopsies from 110 PTC patients and measured the expression of CHI3L1 by RT-qPCR in 14 PTC patients with different degrees of malignancy. The results showed that the mRNA expression of CHI3L1 in malignant PTC groups (obvious invasion and distant metastasis [OIDM], TNM stage T4N1bM1) was significantly higher than that in the healthy control (HC) and relatively benign PTC groups (no metastasis [NM], TNM stage T1N0, T2N0; local metastasis [LM], TNM stage T1N1a, T3N1a, T3bN1b, T4aN1b, T4bN1b) (*P* < 0.05, Fig. 5C). In addition, in the two malignant groups (OIDM and LM), the expression of CHI3L1 in the distant metastasis group was significantly higher than that in the no metastasis group or the lymph node metastasis group (*P* < 0.05, Fig. 5C). We also detected the expression of CHI3L1 in 110 PTC patients by immunohistochemistry; 10 cases of normal or benign thyroid tissue were used as control. CHI3L1 was not expressed in 9 normal or benign thyroid tissues. Its expression was low in 61 patients and high in 40 patients (Fig. 5D; Table 1). Chi-square test analysis showed that the high protein expression of CHI3L1 was significantly associated with T stage (*P* < 0.001), N stage (*P* < 0.001), M stage (*P* = 0.007), gross ETE stage (*P* < 0.001), and age (< 55 vs. ≥ 55, *P* = 0.013) (Table 4). The results suggest that the protein expression of CHI3L1 in PTC is associated with PTC metastasis and that CHI3L1 is an important positive regulator of PTC metastasis.

**Table 1:** The relationship between the expression level of CHI3L1 protein and the Clinical features of 110 patients with PTC

Clinical features		Number of	Expression level of CHI3L1		χ^2^	*P*-value
		cases	Low/None	High		
Sex	Male	23	14(61%)	9(39%)	0.096	0.756
	Female	87	56(64%)	31(36%)		
Age	< 55	74	53(72%)	21(28%)	6.231	0.013
	≥ 55	36	17(47%)	19(53%)		
T stage	T1	29	27(93%)	2(7%)	29.661	< 0.001
	T2	24	18(75%)	6(25%)		
	T3	25	16(64%)	9(36%)		
	T4	32	9(28%)	23(72%)		
N stage	N0	41	39(95%)	2(5%)	29.981	< 0.001
	N1a	11	7(64%)	4(36%)		
	N1b	58	24(41%)	34(59%)		
M stage	M0	101	68(67%)	33(33%)	7.265	0.007
	M1	9	2(22%)	7(78%)		
Gross ETE*	No	51	43(84%)	8(16%)	17.568	< 0.001
	Yes	59	27(46%)	32(54%)		
BRAF^V600E^	Mut	90	58(64%)	32(36%)	0.140	0.709
	Wildtype	20	12(60%)	8(40%)		

* Gross Extrathyroidal extension (ETE): invading the subcutaneous soft tissues, larynx, trachea, esophagus, or recurrent laryngeal nerve (RLN)

### Function of CHI3L1 validated in cell line TPC-1 by expression alternation

**Fig. 6:**
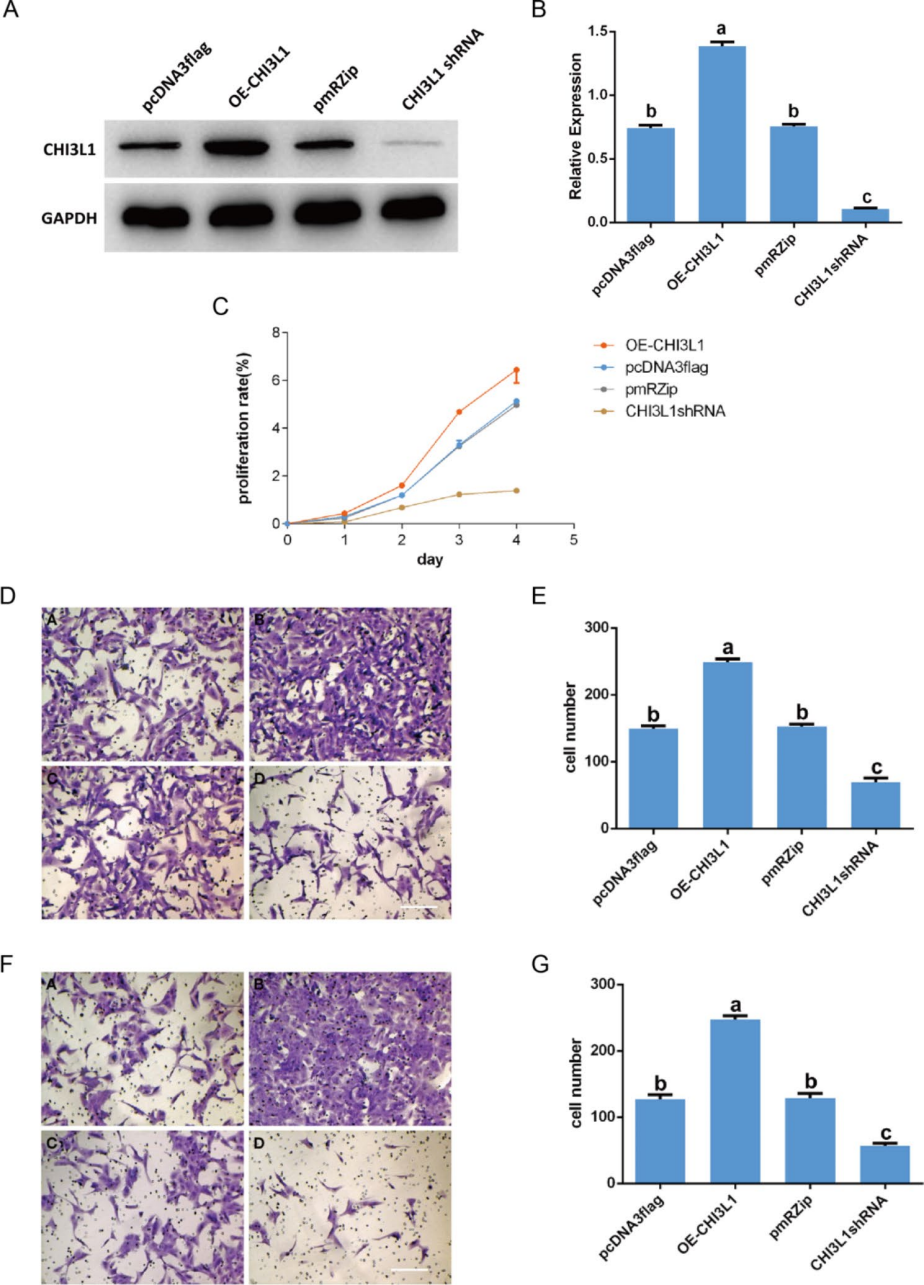
CHI3L1 promotes proliferation, invasion, and metastasis in TPC-1 cells. TPC-1 cells overexpressing CHI3L1 (OE-CHI3L1) or with CHI3L1 knockdown (CHI3L1 shRNA) were included. pcDNA3flag and pmRZip empty plasmid were used as controls. (**A**-**B**) Expression. (**C**) Cell proliferation. (**D**-**E**) Cell invasion. (**F**-**G**) Cell metastasis. Scale bar, 8 μm. In the bar charts, different lowercase letters indicate significant differences (*P* < 0.05)

To investigate the function of CHI3L1 in thyroid cancer cells, TPC-1 cells were transfected with CHI3L1 overexpression vector or infected with shRNA virus (Fig. 6A-B), followed by measurement of cell proliferation, invasion, and metastasis. The results showed that the expression of CHI3L1 (Fig. 6A-B, Table S5), cell proliferation (Fig. 6C, Table S6), cell invasion (Fig. 6D-E, Table S7), and cell metastasis (Fig. 6F-G, Table S8) were significantly increased in cells overexpressing CHI3L1 (OE CHI3L1) and significantly decreased in cells with CHI3L1 knockdown (CHI3L1 shRNA). These results suggest that CHI3L1 is an important regulator for the malignant development of PTC cells, promoting proliferation, invasion, and metastasis in TPC-1 cells in vitro. These results were consistent with our scRNA-seq results and our analysis of clinical biopsies.

## Discussion

PTC is the most common histological type of thyroid cancer [34]. In general, the development of PTC is slow and PTCs are less aggressive than some other malignant cancers [35, 36]. However, lymph node metastasis often occurs in PTC patients, who show regional infiltration, distant metastasis, and treatment tolerance, which tends to lead to high rates of recurrence and poor clinical outcomes [37–39]. In recent years, with the development of scRNA-seq technology, many cellular and molecular mechanisms of carcinogenesis have been elucidated [40], including thyroid cancer [41, 42]. In the current study, the single-cell transcriptomic profiles of PTC1 (with lower malignancy and relatively good prognosis) and PTC2 (with higher malignancy and poorer prognosis) are presented. Importantly, CHI3L1 stood out from a variety of genes related to PTC2, particularly in a subset of epithelial cells. This gene was additionally functionally validated by overexpression and knockdown in a cell line model in vitro, and its expression was further investigated both on the mRNA and the protein level for its universality in a cohort of PTC patients.

CNV is an important molecular mechanism underlying different human diseases, such as cancer [41], genetic disorders [42], and cardiovascular disease [43]. It is generally believed that the occurrence, deterioration, and metastasis of tumors are closely related to CNV [41–44], and the occurrence of CNV in or around tumor-associated gene sequences may initiate the activation of oncogenes, the inactivation of tumor suppressor genes, and finally the occurrence of tumor [45]. In our study, the CNV pattern is inferred from scRNA-seq data, which shows that CNVs almost exclusively present in the epithelial cell cluster. This result verifies that the biopsies are of PTC origin, and that PTC is mainly comprised of malignant epithelial cells. Specifically, current data suggest that PTC is derived from follicular epithelial cells of the thyroid gland.

Differences in CNVs and enriched molecular pathways among the five PTC epithelial cell subsets fully suggest the tumor heterogeneity within PTCs. The results of our pseudotime trajectory analysis of five epithelial cell subsets suggest their possible chronological evolutionary order, and Epi02 seems to be the most important subset of PTC. Importantly, further analysis of the DEGs between PTC1 and PTC2 in the Epi02 subset indicated that CHI3L1 is one of the main DEGs in this cell subset. Our pseudotime trajectory analysis also demonstrated the increased expression of CHI3L1 in Epi02. Therefore, CHI3L1 may play an important role in the invasion and metastasis of PTC, specifically in a subset of epithelial cancer cells.

CHI3L1, also known as cartilage glycoprotein 39 (GP-39), is a member of the glycosidase 18 gene family [46]. CHI3L1 encodes a secreted glycoprotein that is expressed in different types of human cells [33]. We analyzed the CHI3L1 expression profile in the TCGA database. This gene is highly expressed in a variety of tumors, including gastric cancer, lung cancer, and ovarian cancer [47]. It is involved in different signaling pathways of tumor invasion and metastasis, such as epithelial-mesenchymal transition, PI3K-Akt, Ras-MAPK, and TSC-mTOR. A recent report indicated that CHI3L1 protein expression is low or not detected in benign thyroid cancer but high in malignant thyroid cancer, and that higher protein expression levels of CHI3L1 are associated with a higher probability of extrathyroid infiltration and lymph node metastasis and a higher risk of recurrence of PTC [26]. Another report also showed that overexpression of CHI3L1 in PTC is closely related to tumor size, lymph node metastasis, and tumor invasion [25].

In the present study, the scRNA-seq analysis revealed that CHI3L1 is overexpressed in the highly malignant and metastatic PTC patient PTC2 compared to the control. Critically, the analysis at the single-cell level further revealed that CHI3L1 overexpression is mainly observed in the subsets of epithelial cancer cells. We further investigated the expression of CHI3L1 on both the mRNA and the protein level with clinical PTC biopsies of different TNM stages, and confirmed its correlation with PTC invasion and metastasis. The expression of CHI3L1 in 14 PTC biopsies with different degrees of malignancy was evaluated with qRT-PCR, and the results showed that the expression of CHI3L1 is positively correlated with the malignancy degree of PTC. We also performed immunohistochemistry analysis on 110 cases of PTC versus 10 cases of normal or benign thyroid tissue as control, and revealed a significant positive relationship between CHI3L1 protein expression and PTC stage, especially the T, N, and M stages.

Moreover, CHI3L1 is closely associated with a variety of diseases, and it plays a major role in tissue injury, tissue repair, and remodeling responses [41]. Studies have shown that CHI3L1 stimulates cell growth and proliferation of different cells through different signal pathways [48]. In particular, CHI3L1 facilitates the growth of skin and fetal lung fibroblasts through MAPK and Akt signaling [49], and promotes the growth and proliferation of bronchial smooth muscle cells through Akt, ERK, and p38 signaling [50]. Meanwhile, CHI3L1 advances the growth, proliferation, invasion, and metastasis of a variety of cancers [51]. CHI3L1 enhances the proliferation of HEK293 cells, U373 cells, and U87 MG cells through the ERK1/2 signaling pathway [52] and elevates the proliferation and migration of SW480 cells and COLO 205 cells through the NF-κB signaling pathway [53]. CHI3L1 also induces the expression of pro-tumorigenic molecules, including CXCL2, MMP-2, and MMP-9, which contribute to tumor growth and proliferation [54]. Importantly in this research with verity aspects of evidences we have proved that CHI3L1 specifically promotes the malignant development of PTC.

## Conclusions

In summary, the present study described the single-cell profile of PTC and revealed that CHI3L1 was a major positive regulator for the malignant development of PTC, particularly through the epithelial cell subset Epi02. In addition, our experiments showed that CHI3L1 promoted the proliferation, invasion, and metastasis of cell line TPC-1 in vitro. Furthermore, a qRT-PCR analysis of 14 PTC patients showed the positive correlation between the expression of CHI3L1 with the malignancy of PTC, and a cohort study of 110 PTC cases validated the distribution of CHI3L1 in malignant PTCs both on the mRNA and on the protein level. Combining all this evidence, we concluded that CHI3L1 is one of the important genes that regulate the malignant development of PTC. Our findings deepened our understanding of the molecular mechanisms underlying the malignant development of PTC and provide new insights into the clinical diagnosis and treatment of PTC.

## Electronic supplementary material

Below is the link to the electronic supplementary material.

Supplementary Material 1

Supplementary Material 2

Supplementary Material 3

Supplementary Material 4

Supplementary Material 5

Supplementary Material 6

Supplementary Material 7

Supplementary Material 8

Supplementary Material 9

Supplementary Material 10

## Data Availability

The authors confirm that all relevant data are included in the article and that materials are available upon request from the authors. TCR sequencing data reported in this paper was deposited under the following doi: https://www.jianguoyun.com/p/DRp-mmAQotabCxifzOsEIAA. Beside, R code/script files are included in ‘PTC1_PTC2_harmony’ in jianguoyun as HTML document.
