# Supplementary material for: Regulatory role of Chitinase 3-like 1 gene in papillary thyroid carcinoma proved by integration analyses of single-cell sequencing with cohort and experimental validations
Source: Cancer Cell Int. 2023 Jul 21;23:145. doi: 10.1186/s12935-023-02987-7 (PMC10362555; doi:10.1186/s12935-023-02987-7)
Supplement: Supplementary file 1 — Supplementary Material 1 [file 12935_2023_2987_MOESM1_ESM.docx]

Supplementary Material

Regulatory Role of Chitinase 3-Like 1 Gene in Papillary Thyroid Carcinoma Proved by Integration Analyses of Single-cell Sequencing with Cohort and Experimental Validations

1.1 Preparation of single-cell suspensions

Fresh tumor tissues were cut into approximately 1-mm3 pieces in 1 × PBS using scissors. Tissue fragments were enzymatically digested in DMEM containing collagenase I (Solarbio, Cat. no. C8140, 3000 U/mL), collagenase II (Solarbio, Cat. no. C150, 9000 U/mL), and hyaluronidase (Solarbio, Cat. no. H8030) for 30 min at 37°C with a shaking speed of 1000 rpm for about 30 min. The suspended cells were filtered through a 70-µm Cell-Strainer nylon mesh (Falcon, Cat. no. 35235) and centrifuged at 1000 × rpm for 5 min at 4°C. After removing the supernatant, the cell pellets were washed twice with 1 × PBS, and then the cells were stained with 0.4% Trypan blue (Invitrogen, Cat. no. T10282) to check cell viability and diluted with PBS containing 0.04% BSA to about 1 × 106 cells/mL for scRNA-seq.

1.2 Quality control and batch effect correction of scRNA-seq data

The de-multiplexed and aligned data were quantified using CellRanger version 3.01. Quality control filtering was applied to remove any cells for which > 30% of the UMIs were mapped to mitochondrial genes. Cells that contained < 200 or > 8000 genes were considered outliers and also discarded. The Seurat R package (version 4.0.1) was used to normalize individual experiments using the “LogNormalize” method. The CCA method in Seurat was used to overcome batch effects. Firstly, we selected a subset of 2000 genes that were highly variable using the “FindVariableFeatures” option. We then chose the first 20 principal components, which were used for integration. A combined dataset was created by finding anchors between the individual datasets to create a batch-corrected expression matrix.

1.3 Dimensional reduction and cellular annotation

The corrected datasets were then clustered using graph-based clustering. We used Clustree to assess the stability of clusters at a resolution of 0.2 to 1 and determined that a resolution of 0.6 gave the highest number of stable clusters with cells from each donor represented in each cluster. The FindMarkers function identified markers for each cluster by using parameters (log2FC > 0.25 and Padj < 0.05). The clustering results were visualized using t-distributed stochastic neighbor embedding (tSNE) and uniform manifold approximation and projection (UMAP). Cell types were defined by canonical marker genes provided on Cellmarker (http://biocc.hrbmu.edu.cn/CellMarker/) and Panglao DB (<https://panglaodb.se/>).

1.4 Western blot

TPC-1 cells were lysed in lysis buffer (Beyotime, Shanghai, China) to extract total protein. The protein concentrations of the samples were measured with a BCA protein assay kit (Bio-Rad, Hercules, CA, USA). Western blot was performed according to standard protocols. The following antibodies were used in this study: anti-GAPDH (SAB 48102) and anti-CHI3L1 (ab77528).

1.5 Cell proliferation, cell migration and invasion analysis

TPC-1 cells were seeded in 96-well plates at a density of 1 × 103 cells/well and cultured in RPMI-1640 with 10% FBS for 0, 1, 2, 3, and 4 days at 37°C. The CCK8 assay (Beyotime, Cat. No. C0037) was performed according to standard protocols. Cells were incubated with 100 μL CCK-8 (1:10 dilution) at 37°C for 4 h. The optical density at 450 nm was determined. Each sample was analyzed in triplicate.

For the Transwell migration assay, 1 × 105 cells were added to the upper chamber with a serum-free medium, and the lower chamber contained a culture medium with 10% FBS. After 12–48 h of incubation, the cells remaining inside the upper chamber were removed with a cotton swab. All experiments were executed at least three times in triplicate. The inserts were fixed with methanol for 20 min and then stained with 0.25% crystal violet. The invaded cells were counted in five randomly selected microscopic views. Cell invasion was measured using Matrigel-coated Transwell chambers as described previously.
