# Supplementary material for: Regulatory role of Chitinase 3-like 1 gene in papillary thyroid carcinoma proved by integration analyses of single-cell sequencing with cohort and experimental validations
Source: Cancer Cell Int. 2023 Jul 21;23:145. doi: 10.1186/s12935-023-02987-7 (PMC10362555; doi:10.1186/s12935-023-02987-7)
Supplement: Supplementary file 2 — Supplementary Material 2 [file 12935_2023_2987_MOESM2_ESM.docx]

**Table S1.** Clinicopathologic Characteristic of two representative PTC patients

| **Variable** | | **PTC1** | **PTC2** |
| --- | --- | --- | --- |
| Age (years) | | 71 | 63 |
| Sex | | Female | Female |
| Pathology | | PTC | PTC |
| Primary Tumor | Size(diameter, cm) | >10 | >6 |
| Gross  extrathyroidal  extension | Strap muscles | No | Yes |
|  | larynx | No | Yes |
|  | trachea | No | Yes |
|  | esophagus | No | No |
|  | RLN* | No | Yes |
|  | Carotid artery | No | No |
| Lymph node  metastasis | N1a | Yes | Yes |
|  | N1b | Yes | Yes |
|  | Mediastinal | No | Yes |
| Distant  metastasis | Lung | No | Yes |
|  | Bone | No | Yes |
|  | Liver | No | No |
| Radioactive iodine | | Sensitive | Refractory |
| BRAF | | Mutation | Wild |
