# Supplementary material for: Regulatory role of Chitinase 3-like 1 gene in papillary thyroid carcinoma proved by integration analyses of single-cell sequencing with cohort and experimental validations
Source: Cancer Cell Int. 2023 Jul 21;23:145. doi: 10.1186/s12935-023-02987-7 (PMC10362555; doi:10.1186/s12935-023-02987-7)
Supplement: Supplementary file 3 — Supplementary Material 3 [file 12935_2023_2987_MOESM3_ESM.docx]

**Table S4**. The primers sequence used for qRT-PCR

| **Gene name** | **Primer sequence (5'-3')** | |
| --- | --- | --- |
|  | **Forward primer** | **Reverse primer** |
| *CHI3L1* | GTGAAGGCGTCTCAAACAGG | GAAGCGGTCAAGGGCATCT |
| *Actin* | CTCCATCCTGGCCTCGCTGT | GCTGTCACCTTCACCGTTCC |
