# Supplementary material for: Regulatory role of Chitinase 3-like 1 gene in papillary thyroid carcinoma proved by integration analyses of single-cell sequencing with cohort and experimental validations
Source: Cancer Cell Int. 2023 Jul 21;23:145. doi: 10.1186/s12935-023-02987-7 (PMC10362555; doi:10.1186/s12935-023-02987-7)
Supplement: Supplementary file 4 — Supplementary Material 4 [file 12935_2023_2987_MOESM4_ESM.docx]

**Table S5.** Gray analysis by western blotting

| **IOD** | **sample** | **1** | **2** | **3** | **mean±s.d.** | **t-test** |
| --- | --- | --- | --- | --- | --- | --- |
| CHI3L1 | pcDNA3flag | 20345.924 | 19759.853 | 20508.045 | 20204.61±321.38 |  |
|  | OE-CHI3L1 | 38930.823 | 38082.995 | 38676.116 | 38563.31±355.20 |  |
|  | pmRZip | 22951.581 | 22561.51 | 23023.409 | 22845.50±202.94 |  |
|  | CHI3L1shRNA | 2931.296 | 2808.518 | 3160.69 | 2966.83±145.95 |  |
| GAPDH | pcDNA3flag | 26919.468 | 27653.589 | 27241.882 | 27271.65±300.44 |  |
|  | OE-CHI3L1 | 27500.61 | 28253.388 | 27834.267 | 27862.76±307.98 |  |
|  | pmRZip | 29953.125 | 30632.731 | 30194.61 | 30260.16±281.29 |  |
|  | CHI3L1shRNA | 27307.832 | 28093.782 | 27887.075 | 27762.90±332.66 |  |
| CHI3L1/GAPDH | pcDNA3flag | 0.756 | 0.715 | 0.753 | 0.74±0.02 |  |
|  | OE-CHI3L1 | 1.416 | 1.348 | 1.390 | 1.38±0.03 | 0.000 |
|  | pmRZip | 0.766 | 0.737 | 0.763 | 0.76±0.01 | 0.433 |
|  | CHI3L1shRNA | 0.107 | 0.100 | 0.113 | 0.11±0.01 | 0.000 |
