# Supplementary material for: Regulatory role of Chitinase 3-like 1 gene in papillary thyroid carcinoma proved by integration analyses of single-cell sequencing with cohort and experimental validations
Source: Cancer Cell Int. 2023 Jul 21;23:145. doi: 10.1186/s12935-023-02987-7 (PMC10362555; doi:10.1186/s12935-023-02987-7)
Supplement: Supplementary file 5 — Supplementary Material 5 [file 12935_2023_2987_MOESM5_ESM.docx]

**Table S6.** Proliferation rate of TPC-1 cell by CCK-8

| **time** | **sample** | **1** | **2** | **3** | **4** | **5** | **mean±s.d.** | **t-test** |
| --- | --- | --- | --- | --- | --- | --- | --- | --- |
| day0 | pcDNA3flag | 0.12 | -0.01 | -0.02 | -0.05 | -0.04 | 0±0.07 |  |
|  | OE-chi3L1 | 0.02 | 0.06 | 0.05 | -0.07 | -0.07 | 0±0.06 | 1.000 |
|  | pmRZip | 0.03 | 0.07 | 0.06 | -0.08 | -0.08 | 0±0.07 | 1.000 |
|  | chi3L1shRNA | -0.12 | 0.03 | 0.02 | 0.03 | 0.05 | 0±0.07 | 1.000 |
| day1 | pcDNA3flag | 0.25 | 0.36 | 0.34 | 0.25 | 0.32 | 0.3±0.05 |  |
|  | OE-chi3L1 | 0.43 | 0.43 | 0.43 | 0.43 | 0.44 | 0.43±0.01 | 0.004 |
|  | pmRZip | 0.24 | 0.19 | 0.21 | 0.30 | 0.29 | 0.25±0.05 | 0.234 |
|  | chi3L1shRNA | 0.06 | 0.07 | 0.06 | 0.09 | 0.09 | 0.07±0.02 | 0.001 |
| day2 | pcDNA3flag | 1.21 | 1.16 | 1.20 | 1.19 | 1.19 | 1.19±0.02 |  |
|  | OE-chi3L1 | 1.55 | 1.55 | 1.63 | 1.63 | 1.64 | 1.6±0.05 | 0.000 |
|  | pmRZip | 1.16 | 1.17 | 1.16 | 1.25 | 1.22 | 1.19±0.04 | 0.892 |
|  | chi3L1shRNA | 0.59 | 0.65 | 0.70 | 0.72 | 0.73 | 0.68±0.06 | 0.000 |
| day3 | pcDNA3flag | 3.11 | 3.50 | 3.18 | 3.48 | 3.20 | 3.29±0.18 |  |
|  | OE-chi3L1 | 4.64 | 4.62 | 4.71 | 4.72 | 4.72 | 4.68±0.05 | 0.000 |
|  | pmRZip | 3.37 | 3.38 | 3.37 | 3.06 | 3.06 | 3.25±0.17 | 0.731 |
|  | chi3L1shRNA | 1.16 | 1.23 | 1.24 | 1.24 | 1.24 | 1.22±0.04 | 0.000 |
| day4 | pcDNA3flag | 5.12 | 5.13 | 5.10 | 5.13 | 5.17 | 5.13±0.02 |  |
|  | OE-chi3L1 | 6.17 | 5.96 | 6.00 | 7.06 | 7.02 | 6.44±0.55 | 0.005 |
|  | pmRZip | 4.92 | 4.94 | 5.03 | 4.98 | 4.98 | 4.97±0.04 | 0.003 |
|  | chi3L1shRNA | 1.41 | 1.39 | 1.34 | 1.39 | 1.40 | 1.38±0.03 | 0.000 |
