# Supplementary material for: Regulatory role of Chitinase 3-like 1 gene in papillary thyroid carcinoma proved by integration analyses of single-cell sequencing with cohort and experimental validations
Source: Cancer Cell Int. 2023 Jul 21;23:145. doi: 10.1186/s12935-023-02987-7 (PMC10362555; doi:10.1186/s12935-023-02987-7)
Supplement: Supplementary file 6 — Supplementary Material 6 [file 12935_2023_2987_MOESM6_ESM.docx]

**Table S7.** Test of cell migration

| **sample** | **1** | **2** | **3** | **4** | **mean±s.d.** | **t-test** |
| --- | --- | --- | --- | --- | --- | --- |
| pcDNA3flag | 144 | 154 | 151 | 148 | 149.35±4.33 |  |
| OE-chi3L1 | 243 | 254 | 251 | 247 | 248.65±4.56 | 0.000 |
| pmRZip | 152 | 156 | 155 | 148 | 152.69±3.58 | 0.125 |
| chi3L1shRNA | 74 | 71 | 60 | 72 | 69.56±6.43 | 0.000 |
