# Supplementary material for: Regulatory role of Chitinase 3-like 1 gene in papillary thyroid carcinoma proved by integration analyses of single-cell sequencing with cohort and experimental validations
Source: Cancer Cell Int. 2023 Jul 21;23:145. doi: 10.1186/s12935-023-02987-7 (PMC10362555; doi:10.1186/s12935-023-02987-7)
Supplement: Supplementary file 7 — Supplementary Material 7 [file 12935_2023_2987_MOESM7_ESM.docx]

**Table S7.** Test of cell invasion

| **sample** | **1** | **2** | **3** | **4** | **mean±s.d.** | **t-test** |
| --- | --- | --- | --- | --- | --- | --- |
| pcDNA3flag | 128 | 137 | 121 | 123 | 127.46±7.25 |  |
| OE-chi3L1 | 241 | 250 | 244 | 254 | 247.32±5.59 | 0.000 |
| pmRZip | 138 | 123 | 122 | 132 | 128.85±7.53 | 0.819 |
| chi3L1shRNA | 59 | 51 | 60 | 57 | 56.77±4.08 | 0.001 |
