# Supplementary material for: Regulatory role of Chitinase 3-like 1 gene in papillary thyroid carcinoma proved by integration analyses of single-cell sequencing with cohort and experimental validations
Source: Cancer Cell Int. 2023 Jul 21;23:145. doi: 10.1186/s12935-023-02987-7 (PMC10362555; doi:10.1186/s12935-023-02987-7)
Supplement: Supplementary file 8 — Supplementary Material 8 [file 12935_2023_2987_MOESM8_ESM.docx]

**Table S7.** Expression of *CHI3L1* in PTC clinical biopsies with different degrees of malignancy by RT-qPCR

|  | Δ | base level | #VALUE! | #VALUE! |
| --- | --- | --- | --- | --- |
| HC group | | | | |
| CN1-1 | 12.15 | 12.15 | 0 | 1 |
| CN1-2 | 12.21 | 12.15 | 0.06 | 0.959264119 |
| CN1-3 | 12.07 | 12.15 | -0.08 | 1.057018041 |
| CN2-1 | 10.76 | 12.15 | -1.39 | 2.620786808 |
| CN2-2 | 10.67 | 12.15 | -1.48 | 2.789487333 |
| CN2-3 | 10.85 | 12.15 | -1.3 | 2.462288827 |
| NM group | | | | |
| CT1-1 | 7.34 | 12.15 | -4.81 | 28.05138308 |
| CT1-2 | 7.22 | 12.15 | -4.93 | 30.48441594 |
| CT1-3 | 7.06 | 12.15 | -5.09 | 34.05984584 |
| CT2-1 | 5.89 | 12.15 | -6.26 | 76.6386371 |
| CT2-2 | 5.94 | 12.15 | -6.21 | 74.02804377 |
| CT2-3 | 5.83 | 12.15 | -6.32 | 79.89315513 |
| LM group | | | | |
| N1-1 | 6.04 | 12.15 | -6.11 | 69.07060714 |
| N1-2 | 6.17 | 12.15 | -5.98 | 63.11889309 |
| N1-3 | 6.37 | 12.15 | -5.78 | 54.94818793 |
| N2-1 | 4.51 | 12.15 | -7.64 | 199.4661324 |
| N2-2 | 4.78 | 12.15 | -7.37 | 165.4211623 |
| N2-3 | 4.63 | 12.15 | -7.52 | 183.5462717 |
| N3-1 | 3.12 | 12.15 | -9.03 | 522.7582084 |
| N3-2 | 3.24 | 12.15 | -8.91 | 481.0356476 |
| N3-3 | 3.16 | 12.15 | -8.99 | 508.4633577 |
| N4-1 | 6.33 | 12.15 | -5.82 | 56.49299176 |
| N4-2 | 5.67 | 12.15 | -6.48 | 89.26359465 |
| N4-3 | 5.63 | 12.15 | -6.52 | 91.77313587 |
| N5-1 | 3.33 | 12.15 | -8.82 | 451.9439341 |
| N5-2 | 3.51 | 12.15 | -8.64 | 398.9322648 |
| N5-3 | 3.21 | 12.15 | -8.94 | 491.1432291 |
| OIDM group | | | | |
| M1-1 | 6.04 | 12.15 | -6.11 | 69.07060714 |
| M1-2 | 6.5 | 12.15 | -5.65 | 50.21338227 |
| M1-3 | 5.82 | 12.15 | -6.33 | 80.44885597 |
| M2-1 | 3.55 | 12.15 | -8.6 | 388.023441 |
| M2-2 | 3.67 | 12.15 | -8.48 | 357.0543786 |
| M2-3 | 3.5 | 12.15 | -8.65 | 401.7070581 |
| M3-1 | 3.58 | 12.15 | -8.57 | 380.0380341 |
| M3-2 | 3.46 | 12.15 | -8.69 | 413.0005807 |
| M3-3 | 3.34 | 12.15 | -8.81 | 448.8221293 |
| M4-1 | 3.43 | 12.15 | -8.72 | 421.6786008 |
| M4-2 | 3.47 | 12.15 | -8.68 | 410.1477773 |
| M4-3 | 3.15 | 12.15 | -9 | 512 |
| M5-1 | 2.68 | 12.15 | -9.47 | 709.1760477 |
| M5-2 | 2.54 | 12.15 | -9.61 | 781.444715 |
| M5-3 | 2.4 | 12.15 | -9.75 | 861.0779292 |

HC：healthy control;

NM：no metastasis (TNM stage T1N0, T2N0);

LM：general metastasis (TNM stage T1N1a, T3N1a, T3bN1b, T4aN1b, T4bN1b);

OIDM：obvious invasion and distant metastasis (TNM stage T4N1bM1)
