# Supplementary material for: Regulatory role of Chitinase 3-like 1 gene in papillary thyroid carcinoma proved by integration analyses of single-cell sequencing with cohort and experimental validations
Source: Cancer Cell Int. 2023 Jul 21;23:145. doi: 10.1186/s12935-023-02987-7 (PMC10362555; doi:10.1186/s12935-023-02987-7)
Supplement: Supplementary file 10 — Supplementary Material 10 [file 12935_2023_2987_MOESM10_ESM.docx]

**Table S3**. The primers sequence of *CHI3L1* shRNA vector

| *CHI3L1* | **Primer sequence (5'-3')** |
| --- | --- |
| **Forward primer** | GATCCGCATGCTCAACACACTCAAGACTCGAGTCTTGAGTGTGTTGAGCATG |
| **Reverse primer** | AATTCAAAAAGCATGCTCAACACACTCAAGACTCGAGTCTTGAGTGTGTTG |
